# Pharmacokinetic-pharmacodynamic profiling of four antimicrobials against Gram-negative bacteria collected from Shenyang, China

**DOI:** 10.1186/1471-2334-10-171

**Published:** 2010-06-15

**Authors:** Yun Zhuo Chu, Su Fei Tian, Bai Yi Chen, Hua Nian, Hong Shang, Guo Quan Sun

**Affiliations:** 1 Department of Laboratory Medicine, The First Hospital of China Medical University, Shenyang, 110001, China; 2 Division of Infectious Diseases, The First Hospital of China Medical University, Shenyang, 110001, China

## Abstract

**Background:**

To examine common antimicrobial regimens used in eradicating certain nosocomial Gram-negative pathogens and determine which ones are likely to be the most suitable as empirical choices in Shenyang, China.

**Methods:**

A 5000-subject Monte Carlo simulation was conducted to determine the cumulative fraction of response (CFR) for meropenem, imipenem, cefepime, piperacillin/tazobactam and levofloxacin against *Escherichia coli, Klebsiella pneumoniae, Enterobacter cloacae, Acinetobacter baumannii *and *Pseudomonas aeruginosa *collected in 2006 and 2007 from Shenyang.

**Results:**

Meropenem and imipenem had the highest CFRs against the *Enterobacteriaceae *(97%-100%), followed by cefepime. No antibiotic simulated regimen achieved optimal CFR against *P. aeruginosa *and *A. baumannii*. Piperacillin/tazobactam dosed at 4.5 g q8h achieved the lowest CFR against all bacteria.

**Conclusions:**

This study suggests that the carbapenems provide the greatest likelihood of clinical success for the *Enterobacteriaceae*, and combination therapy might be needed when choosing empirical therapy, especially when *A. baumannii *or *P. aeruginosa *are suspected.

## Background

The rapid increase in the prevalence of multidrug resistant microorganisms has threatened the physician's ability to treat serious infections. Since there are a limited number of antimicrobials available that can treat multidrug resistant gram-negative bacteria such as Enterobacteriaceae harboring extended-spectrum β-lactamases (ESBLs), multidrug-resistant *P. aeruginosa *and *A. baumannii*, the best approach to combating resistance and providing effective therapy is optimizing the use of currently available antimicrobial agents.

Antibiotic surveillance studies lack consideration of pharmacokinetics and provide little information about optimal dosing. The consideration of minimum inhibitory concentration (MIC) distribution, antibiotic regimen and pharmacokinetic parameter derived from human studies via application of pharmacokinetic/pharmacodynamic (PK/PD) models with Monte Carlo simulation offers a more useful tool for clinicians to predict successful outcomes [1].

Given that Gram-negative bacteria resistance is increasing rapidly and varies considerably by geographical location in China, location-specific information on pharmacokinetic-pharmacodynamic profiling of common antibiotics (such as meropenem, imipenem, cefepime, piperacillin/tazobactam and levofloxacin) against problem pathogens is of particular concern, thus we conducted this study to assess common antibiotic regimens utilized in Shenyang in order to provide insight into the appropriate antibiotic and dosing options for the empiric therapy of common nosocomial pathogens.

## Methods

The present study was performed with isolates collected consecutively during 2006 to 2007 from patients hospitalized in The First Hospital of China Medical University, The Second Hospital of China Medical University, and Provincial People Hospital in Shenyang. Included bacterial strains were isolates of non-duplicate *E. coli *(n = 414), *K. pneumoniae *(n = 236), *E. cloacae *(n = 40), *P. aeruginosa *(n = 281) and *A. baumannii *(n = 115). Identification of the species level was performed by each laboratory of the participant Hospital with either conventional biochemical methodology or an automated system (Vitek 2, bioMérieux, France).

The MIC data of meropenem, imipenem, cefepime, piperacillin/tazobactam and levofloxacin against these bacteria were determined by the agar dilution method at Department of Laboratory Medicine, The First Hospital of China Medical University according to Clinical and Laboratory Standards Institute guidelines (Table 1). Control strains of *E. coli *(ATCC 25922) and *P. aeruginosa *(ATCC 27853) were run with each set of MIC determinations.

**Table 1:** MIC distributions for various antimicrobials tested from Shenyang in China

Species(no.) or antibiotic	%S	% of isolates susceptible at MIC(μg/ml)																MIC_50_	MIC_90_	MIC range
		0.006	0.01	0.03	0.06	0.125	0.25	0.5	1	2	4	8	16	32	64	128	256			
*E.coli *(414)																				
MEM	99.8	94.4	1.7	1.5	1	0.5	0.7	0	0	0.2	0	0	0	0	0	0	0	0.03	0.032	.03 - 8
IPM	99.8	17.9	11.5	21.5	43.3	4.6	0.5	0.5	0	0.2	0	0	0	0	0	0	0	0.125	0.25	.03 - 16
FEP	69.4	12.7	1.3	2.5	4.1	16.7	5.3	2.7	9	15.3	12.1	8.3	4.4	2.4	1.7	1.5	0	4	32	.03 - 512
TZP	70.5	0.5	0	0	0.2	0.5	1	7.9	19	24.1	16.9	10.2	9.9	3	2.2	4.2	0	8	64	.03 - 512
LVX	30.8	6.8	3	1.2	8.2	5.7	5	1	7.4	18.6	23.3	14.4	5	0.2	0.2	0	0	8	32	.03 - 256
*K.pneumoniae *(236)																				
MEM	99.6	4.7	50.4	34.8	4.9	1.8	0.4	0.9	1.3	0.4	0	0.4	0	0	0	0	0	0.03	0.125	.015 - 16
IPM	97.8	0	0	0.6	13.1	42.2	15.5	2.5	14.9	11.2	0	0	0	0	0	0	0	0.25	2	.03 - 32
FEP	62.3	0	3	13	15.2	3.9	3.5	3.5	7.8	8.2	4.3	10.4	9.5	4.3	4.3	1.3	7.8	4	128	.03 - 512
TZP	44.6	0	0	0	0	0	0	0	1.4	11.6	19.2	12.5	21	18.3	6.7	7.1	2.2	32	128	2 - 512
LVX	64.5	0	23.8	7.9	2.6	3.9	4.8	11	11	4.8	7	8.8	7.5	5.7	1.3	0.4	0	1	32	.03 - 256
*E.cloacae *(40)																				
MEM	97.5	0	0	90	0	0	5	2.5	0	0	0	0	2.5	0	0	0	0	0.03	0.032	.03 - 16
IPM	97.5	0	0	12.5	50	0	17.5	7.5	10	0	0	2.5	0	0	0	0	0	0.06	1	.03 - 8
FEP	67.5	0	0	27.5	7.5	7.5	2.5	0	10	2.5	7.5	2.5	10	12.5	5	2.5	2.5	1	32	.03 - 256
TZP	60	0	0	0	0	0	0	0	2.5	10	22.5	7.5	17.5	7.5	2.5	5	25	16	256	1 - 512
LVX	70	0	0	57.5	7.5	0	2.5	0	2.5	0	2.5	2.5	7.5	15	2.5	0	0	0.03	32	.03 - 64
*P.aeruginosa *(281)																				
MEM	56.8	0	0	1.5	4.1	7	10	13	9.2	4.4	7.4	12.5	20.3	4.4	3	2.9	0	4	32	.03 - 128
IPM	46.5	0	0	1.1	1.1	0.4	0.7	4.1	18	10	11.4	14.8	8.5	19.9	5.2	3	2.1	8	64	.03 - 512
FEP	45.9	0	0	1.5	0	0	0	1.1	6.3	8.5	9.6	18.9	20.7	11.9	8.1	7.8	5.6	16	128	.03 - 512
TZP	43.7	0	0	0.4	0	0	0	0.7	0	1.5	3.7	12.2	8.9	6.7	9.6	10	46	128	256	.03 - 512
LVX	56.1	0	0	5.9	1.1	9.6	15.1	8.9	11	4.4	7.7	14.8	11.4	5.5	2.6	1.5	0.4	1	16	.03 - 256
*A.baumannii *(115)																				
MEM	37.8	0	0	4.2	7.2	5.2	6.1	7.1	6.1	1	1	2	5.1	30.6	11.2	12.2	1	32	128	.03 - 256
IPM	35.7	0	0	2	7.1	8.2	3.1	4.1	6.1	4.1	1	5.1	8.2	23.5	21.4	5.1	1	32	64	.03 - 256
FEP	19.8	0	0	0	0	0	1.9	0	2.7	3.6	8.1	3.6	16.2	7.2	9	22.5	25	64	256	.25 - 512
TZP	17.3	0	0	2	0	0	1.1	0	0	2	0	8.2	4.1	0	1	3.1	79	256	256	.03 - 512
LVX	48.7	0	0	14.8	0.9	2.6	0.9	3.5	3.5	22.6	27	12.2	7	5	0	0	0	4	16	.03 - 32

MEM Meropenem; IPM Imipenem; FEP Cefepime; TZP Piperacillin/tazobactam; LVX Levofloxacin

The percent of the dosing interval during which free (i.e. unbound) drug was above the MIC (% *f *T > MIC) was selected as the pharmacodynamic exposure of interest. The following antibiotic regimens were chosen based on the most common regimens used in Shenyang to treat these suspected pathogens modeled as 30-min intravenous (i.v.) infusions: meropenem 0.5 g every 6 hours (q6h), 1 g q8h, and 1 g q12h; imipenem 0.5 g q6h, 1 g q8h and 1 g q12h; cefepime 1 g q8h and 2 g q8h and 2 g q12h; and piperacillin/tazobactam 4.5 g q8h. A one-compartment i.v. -infusion equation was used to calculate % *f *T > MIC for the β - lactams at steady state as previously described [2]:

where *Ln *is the natural logarithm, *f *is the fraction of unbound drug, *Vd *is the volume of distribution in liters at steady state, *CL_T _*is the total body clearance in liters per hour, and *DI *is the dosing interval for the regimen.

Pharmacokinetic-pharmacodynamic exposures for levofloxacin were measured by calculation of the total drug 24-h area under the concentration-time curve (AUC) to MIC ratio (AUC/MIC). Total drug AUC for levofloxacin regimens (levofloxacin 0.5 every 24 h) were calculated by dividing the daily dose by total plasma clearance

Pharmacokinetic data were obtained from previously published studies with healthy volunteers using previously described selection criteria [2,3]. Table 2 gives the mean and standard deviation for the total body clearance (total body plasma clearance was calculated as CL = dose/AUC, AUC was determined by the trapezoidal rule and was extrapolated to infinity [4]) in liters per hour resulting assumptions used to model total plasma clearance (CL_T_), volume of the central compartment (*V_d_*), fraction unbound and AUC_0-24_.

**Table 2:** Assumptions for pharmacokinetic parameters used during simulations

Antibiotic	Pharmacokinetic (mean ± SD)				Reference
	CL_T _(liters/h)	V_d _(liters)	Fraction unbound (%)	AUC_0-24_	
Meropenem	14.4 ± 1.8	18.6 ± 3.0	0.85-0.98	-	2
Imipenem	10.5 ± 1.4	15.3 ± 3.3	0.80-0.95	-	2
Cefepime	5.3 ± 0.6	13.6 ± 2.4	0.8-0.9	-	2
Piperacillin/tazobactam	10.9 ± 1.2	11.9 ± 1.7	0.65-0.75	-	2
Levofloxacin	-	-	-	48 ± 8	3

A 5000-patient Monte Carlo simulation (Crystal Ball 2000; Decisioneering Inc., Denver, CO) was conducted to calculate estimates of % *f *T > MIC or AUC/MIC ratio for each antibiotic dosage regimen and bacterial population combination. Five thousand estimates of pharmacodynamic exposure were generated for each antibiotic regimen against each bacterial population using values for *CL_T_, Vd, f*, and MIC based on probability distributions, as previously described [2]. During simulations, pharmacokinetic parameters were assumed to follow log-Gaussian distributions and the fraction unbound *f *followed a uniform distribution. Discrete MIC distributions were built for each population of bacteria based on the MIC frequencies, whereby the percentage of bacteria for which each MIC applies is treated as a frequency and values in between the MIC do not exist. The cumulative fraction of response (CFR) was calculated over the MIC distributions using weighted summation [5]. For comparative purpose, bactericidal pharmacodynamic breakpoints were defined as 40% *f *T > MIC for meropenem and imipenem, 50% *f *T > MIC for cefepime, and piperacillin-tazobactam, and a total drug AUC/MIC ≥ 125 for levofloxacin. A regimen that achieved > 90% CFR against a population of organisms was considered optimal [6].

## Results

The CFRs for each antibiotic dosage regimen against each population of bacteria are given in Table 3. Overall, CFRs were highest against the *Enterobacteriaceae*, followed by *P. aeruginosa *and then *A. baumannii*.

**Table 3:** Bactericidal cumulative fractions of response (CFRs) for antibiotic dosage regimens against *Escherichia coli, Klebsiella pneumoniae, Enterobacter cloacae, Pseudomonas aeruginosa *and *Acinetobacter baumannii *collected from Shenyang in China

Regimen	Bactericidal CFR (%)				
	*E. coli*	*K. pneumoniae*	*E. cloacae*	*P. aeruginosa*	*A. baumannii*
Meropenem 0.5 g q6h	100	100	100	52	59
Meropenem 0.5 g q8h	100	100	97	46	59
Meropenem 1 g q8h	100	100	100	54	59
Imipenem 0.5 g q6h	100	99	97	45	36
Imipenem 0.5 g q8h	100	97	97	36	34
Imipenem 1 g q8h	100	99	98	49	37
Cefepime 1 g q8h	90	73	68	48	22
Cefepime 2 g q8h	95	83	78	68	37
Cefepime 2 g q12h	90	74	68	45	22
Piperacillin/tazobactam 4.5 g q8h	71	33	34	11	8
Levofloxacin 0.5 g every 24 h	30	44	68	33	19

Against the *Enterobacteriaceae*, meropenem and imipenem at all simulated dosage regimens achieved the highest CFRs. Because lower doses of meropenem and imipenem (0.5 g q8h or q6h) by the standard 30-minute infusion obtained 97%-100% CFR against *Enterobacteriaceae*, increasing the dose of meropenem to 1 g q8h had no clinical significance for these three species (98%-100% CFR) (Table 3).

Against *E. coli*, cefepime also fared well, with higher CFRs (95%) than the optimal CFR (90%) at the simulated regimen of 2 g q8h and slightly lower CFRs (90%) at the lower dose of 1 g q8h and 2 g q12h. Piperacillin/tazobactam dosed at 4.5 g q8h achieved the lowest CFR against all bacteria. Particularly, levofloxacin regimens achieved significantly less exposure against *E. coli *(30%) than *E. cloacae *(68%) and *K. pneumoniae *(44%).

No antibiotic simulated regimen achieved high enough CFRs against the nonfermenters to warrant its use empirically as monotherapy. Against *P. aeruginosa *and *A. baumannii*, all antibiotic regimens achieved very low CFRs (≤ 60%) with the exception of cefepime dosed at 2000 mg every 8 hours against *P. aeruginosa *(68%).

## Discussion

Our simulations demonstrated that meropenem and imipenem achieved a high likelihood of bactericidal activity against the *Enterobacteriaceae*, with CFRs close to 100%. These findings support other studies [2,7,8] indicating that for mild to moderate infections caused by pathogens with inherently low MICs, such as the *Enterobacteriaceae*, meropenem and imipenem 0.5 g q8h as a 30-minute infusion may be as effective as standard therapy (1 g, q8h), resulting in decreased costs; whereas for treatment of more severe infections and higher risk of *P. aeruginosa *or other antibiotic-resistant, gram-negative pathogens, meropenem at higher dose (2 g, q8h) would optimize the pharmacodynamic parameter of %T > MIC.

Additionally, larger or more frequent doses of antibiotic against the nonfermenters resulted in increases in the CFRs, but not enough to justify the use of any of these agents empirically as monotherapy. This may have clinical relevance, since dosage increase is a common response to try to curb resistance. The data shown here clearly demonstrate that for the regimens evaluated, dosage increase would not be a good option for empiric treatment. Instead, because no single regimen had high CFR against *A. baumannii *and *P. aeruginosa*, the use of empiric combination therapy to treat these pathogens may be worthwhile. A recent study demonstrated that the use of combination antimicrobial therapy for the *P. aeruginosa *bacteremia resulted in an improvement in 30-day survival; at least until the antibiotic susceptibility results were available to help guide therapy [8].

In addition, being different from other study [9], the results of these simulations are strongly supported by the dramatically increasing resistance emerging in carbapenem-resistant *A. baumannii *in Shenyang (China), with only 38%- 36% susceptible to meropenem and imipenem respectively (data not shown). Other study suggests that the carbapenemase *blaOXA-23 *and the AdeABC efflux pump were the importance mechanism of imipenem-resistance in this region (this was done in a separate project). It alludes to the fact that it is critical to know local trends in resistance and MIC distributions, since information from elsewhere (even within the same country) could lead one to utilize less optimal dosing regimens. Ideally, the use of hospital- or unit-specific (i.e. ICU) MIC distributions in those Monte Carlo simulations would provide the most reliable data for designing empirical dosing regimens [10].

There are a few issues that require discussion. With regard to the pharmacokinetic data, the parameters chosen were selected from healthy adults rather than patients. This is due to the lack of comparable pharmacokinetic trials of all these agents in the same patient population; we utilized healthy volunteer data to make a conservative estimate, similar to other studies [2,9]. Additionally, some studies showed that the pharmacodynamic target attainment calculated with healthy subject pharmacokinetic data was predictive of patient target attainment for the β-lactams [11].

Additional limitations of the study are that it does not look at the new carbapenem, doripenem, and neither does it evaluate prolonged nor continuous infusion regimens.

## Conclusions

This pharmacokinetic-pharmacodynamic simulation provides a useful tool that complements susceptibility data to help in the selection of appropriate empirical antibiotic therapy on the regional level. It suggests that the carbapenems, and specifically meropenem, remain the most potent agents for the *Enterobacteriaceae*, and owing to high antibiotic MICs among many Gram-negative rods in Shenyang, combination therapy might be needed when choosing empirical therapy, especially when *A. baumannii *or *P. aeruginosa *are suspected.

## Conflict of interests

The authors declare that they have no competing interests.

## Authors' contributions

YZC and SFT have made substantial contributions to conception and design, or acquisition of data, or analysis and interpretation of data. HN and GQS have been involved in drafting the manuscript. HS conceived of the study, and participated in its design and coordination. BYC carried out the revision for important intellectual content. All authors read and approved the final manuscript.

## Pre-publication history

The pre-publication history for this paper can be accessed here:

http://www.biomedcentral.com/1471-2334/10/171/prepub
